# Primary and secondary breast angiosarcoma: single center report and a meta-analysis

**DOI:** 10.1007/s10549-019-05432-4

**Published:** 2019-09-14

**Authors:** Yara Abdou, Ahmed Elkhanany, Kristopher Attwood, Wenyan Ji, Kazuaki Takabe, Mateusz Opyrchal

**Affiliations:** 1 Department of Medical Oncology, Roswell Park Comprehensive Cancer Center, Buffalo, NY 14203 USA; 2 Department of Statistics, Roswell Park Comprehensive Cancer Center, Buffalo, NY 14203 USA; 3 Department of Surgical Oncology, Roswell Park Comprehensive Cancer Center, Buffalo, NY 14203 USA

**Keywords:** Primary angiosarcoma, Secondary angiosarcoma, Breast angiosarcoma, Stewart–Treves syndrome, Meta-analysis, Case–control study

## Abstract

**Background:**

Primary and secondary breast angiosarcoma is a rare and aggressive malignancy with limited published literature. Optimal management is mostly based on expert opinion. Our study aims to describe a single institution experience with breast angiosarcoma and evaluate other publications on this topic to further clarify prognostic outcomes and treatment modalities in this disease.

**Methods:**

Twenty two cases of breast angiosarcoma from Roswell Park Comprehensive Cancer Center were retrospectively analyzed. Additionally, a systemic review and meta-analysis was conducted to study the association between survival outcomes, overall survival (OS), and recurrence-free survival (RFS) in both primary (PAS) and secondary breast angiosarcoma (SAS).

**Results:**

9 PAS patients (41%) and 13 SAS patients (59%) were retrospectively analyzed. No significant differences were noted in tumor characteristics and survival outcomes between PAS and SAS. Treatment modality had no significant effects on survival outcomes although adjuvant chemotherapy demonstrated a trend towards improved RFS in high grade tumors. 380 PAS and 595 SAS patients were included in the outcome meta-analysis. Survival outcomes were significantly worse with high grade tumors and tumor size of > 5 cm. Adjuvant radiation therapy demonstrated significantly better RFS, while adjuvant chemotherapy had no effect on survival outcomes.

**Conclusion:**

Tumor size and grade seem to be reliable predictors of survival in both PAS and SAS. Mastectomy does not seem to be adding any additional benefit to BCS. Adjuvant radiation therapy showed statistically significant RFS benefit, while adjuvant chemotherapy can be beneficial in high grade tumors.

## Background

Breast sarcomas are a heterogenous group of tumors, accounting for < 1% of all breast malignancies and < 5% of all soft tissue sarcomas [1]. With an annual incidence of 4.6 cases per million, it is considered an extremely rare type of malignancy [2]. Angiosarcomas of the breast account for < 1% of all of soft tissue tumors, but represent the major histological subtype of all breast sarcomas [3]. Despite mimicking breast adenocarcinoma clinically, breast angiosarcoma is a more aggressive malignant tumor of the vascular endothelium, with rapid proliferation and infiltration into surrounding connective tissues and is associated generally with worse prognosis [4, 5]. Based on the etiology, breast angiosarcoma can be divided into two categories: primary (de novo) and secondary (therapy-related).

Breast primary angiosarcoma (PAS) develops de novo with no prior breast radiation. It typically occurs in younger females between ages of 30 to 50, and often presents as a large mass that arises within the breast parenchyma, typically without any skin changes [6]. Secondary angiosarcoma (SAS) has been traditionally linked to two clinical scenarios. More commonly, SAS occurs in the setting of radiation therapy typically given for treatment of breast cancer [7]. SAS can also occur in the setting of chronic lymphedema typically post breast surgery and lymph node dissection (also called Stewart–Treves syndrome) [8]. Typical presentation of SAS is ecchymotic skin lesions with or without ulceration [6], appearing on average 6 years following radiation to the breast and/or chest wall, and occurring mostly in older females between ages 60 and 70. While the incidence of SAS secondary to lymphedema has significantly decreased due to improved surgical techniques [6], the incidence of SAS secondary to radiation therapy has been increasing with the increased use of breast conservation surgical approach [6]. On FISH analysis, SAS exhibits frequent amplification of *cMyc* and *FLT4*, not usually seen with PAS [9]. Furthermore, compared to SAS of the breast, patients with PAS historically have had significantly longer survival, and are less prone to regional recurrence and distant metastasis [10]. Previous studies have demonstrated the role of grade in prognosis [11]. Grading is based on patterns of growth, histological markers of atypia and proliferation indices. They are classified into low, moderate and high grade, with SAS having higher percentage of high grade tumors as compared to PAS [6]. Staging is based on AJCC guidelines, and follows other soft tissue sarcomas; it incorporates both tumor grade as well as size, nodal involvement and presence or absence of distant metastasis.

Due to rarity of these tumors, most available literature consists of case reports and single institution retrospective cohorts, with very limited number of published studies. Previous meta-analysis was published which only included cases of radiation associated SAS diagnosed in European countries [7]. With lack of prospective studies and limited retrospective data optimal management is based on expert opinion, mostly based from experience with other soft tissue sarcomas. Currently, PAS and SAS are managed similarly, with surgical excision being the most common frontline management. Complete resection with optimal margins (R0 resection) is the goal of surgical intervention. The best surgical method of resection remains unclear, with lack of long-term outcome data comparing breast conservative surgery (BCS) versus mastectomy. Role of radiotherapy and chemotherapy remains unclear [12].

In this report, we are describing a single institution experience with both types of breast angiosarcoma, detailing tumor characteristics, treatment, and survival outcomes. We also performed a meta-analysis to update prognostic factors and treatment modalities in this rare population of patients, evaluating treatment modalities including surgery, radiation, and chemotherapy.

## Methods

### Single center retrospective study

This was a retrospective, single center study of patients with histologically proven angiosarcoma of the breast or chest wall seen at Roswell Park Comprehensive Cancer Center (RPCCC) between 1990 and 2015. The study was approved by the local institutional review board, and the requirement for informed consent was waived because of the retrospective nature of this study. Patients were identified through a comprehensive search of the cancer registry at our institution.

Definition of primary angiosarcoma (PAS) versus secondary angiosarcoma (SAS) was in accordance with WHO definition [13]. We also followed the criteria for SAS by Cahan et al. [14] and Arlen et al. [15], in which SAS is defined as angiosarcoma that arises within a previously irradiated field, with a latency period of at least 3 years between radiotherapy and the development of the angiosarcoma, and a histological distinction between the primary cancer and the SAS.

### Statistical analysis

Patient demographic (age and race) and clinical characteristics (prior breast radiation exposure history, prior surgical history, treatment details, and tumor characteristics) were summarized and categorized by primary versus secondary angiosarcoma. Nominal variables were compared using Chi square test. Continuous variables were compared using the Mann-Whitney *U* test. Recurrence-free survival and overall survival were calculated using univariate Kaplan-Meier analysis with log-rank statistics. Analyses were conducted in SAS v9.4 (Cary, NC) at a significance level of 0.05.

### Meta-analysis

#### Literature search strategy

A literature search was performed on SCOPUS and EMBASE electronic databases to identify all studies reported on breast angiosarcoma, between January 2000 and December 2018. Preferred Reporting Items for Systematic Reviews and Meta-Analyses (PRISMA) guidelines were followed for article search and reporting. Computer-based search used keywords such as “Breast” and “angiosarcoma” in the subject heading or title.

#### Eligibility criteria

Studies were included if they contained outcome data on a minimum of two patients with primary or secondary breast angiosarcoma. Non-English literature and papers lacking outcome information were excluded.

#### Data extraction

Two investigators (YA and AE) independently screened all recognized articles from the search results. Relevant information from each study was collected including number of cohorts, duration of data collection, type of angiosarcoma, median tumor size, histological grade, median follow up, median time from exposure to RT until progression, median overall survival (OS), and median disease-free survival (DFS, or similar metric).

#### Statistical analysis

A meta-analysis was conducted on the association between the survival outcomes (overall and recurrence-free survival) and each covariate. For each covariate, the relevant studies were combined using the standard random effects model, from which estimates of the pooled hazard ratio were obtained with 95% confidence intervals. Results were displayed graphically using forest plots. To assess the heterogeneity of the study outcomes, the *Q* and *I*^2^ statistics were examined.

These analyses were applied to the SAS and PAS cohort separately, and then the combined cohort. All analyses were conducted in SAS v9.4 (Cry, NC) at a significance level of 0.05.

## Results

### Single center retrospective study

A total of 12,155 breast cancer patients were seen at RPCCC between 1990 and 2015. Out of these patients, 22 patients were identified with breast angiosarcoma, 9 (41%) patients were diagnosed with PAS, and 13 (59%) patients with SAS. We calculated point prevalence of angiosarcoma at 1.8 per each 1000 breast cancer cases, based on a ratio of 22 cases in our cohort of 12,155 breast cancer patients, which is a similar rate to what was reported in earlier reports [16].

The distributions of clinicopathologic factors in all 22 patients are summarized in Table 1. Patients with PAS had a lower median age compared to SAS patients; 45 years versus 71 years respectively (*p* < 0.001). Out of the total 22 patients, 17 (77%) were white and 5 (23%) were African American. There were no differences in race between PAS and SAS patients. Regarding tumor characteristics, the majority of tumors were higher grade (G3) in both PAS (45%) and SAS (76%). The median tumor size at presentation was 6.9 cm. PAS tumors were slightly bigger in size at 7.3 cm, compared to SAS tumors at 6.9 cm (*p* = 0.93). Multifocal disease was seen in 5 patients with SAS (23%), but none in PAS group. Prior exposure to radiation therapy was present in all 13 patients who developed SAS, with median latency period (time between last exposure to radiation and development of SAS) of 7.8 years. One patient in SAS group had concomitant lymphedema.

**Table 1:** Patient characteristics by diagnosis

Patient and tumor characteristics	PAS *N* (%)	SAS *N* (%)	Overall	*p* value
Number of patients	9 (41)	13 (59)	22 (100)	
Age				
Median age in years	45	71	66	< 0.001
≤ 65	8 (89)	2 (15)	10 (46)	0.002
> 65	1 (11)	11 (85)	12 (54)	
Race				
White	7 (78)	10 (77)	17 (77)	1.00
Black	2 (22)	3 (23)	5 (23)	
Grade				
Low	2 (22)	1 (8)	3 (14)	0.336
Intermediate	2 (22)	1 (8)	3 (14)	
High	4 (45)	10 (76)	14 (64)	
Unknown	1 (11)	1 (8)	2 (9)	
Tumor size (cm)				
Median size in cm	7.3	6.9	6.9	0.931
≤ 6.9	3	3	6	1.00
> 6.9	3	2	5	
Multifocality				
Yes	0	5	5	0.124
No	6	5	11	
Unknown	3	3	6	
Lymphedema				
Yes	0	1	1	1.00
No	9	9	18	
Time from prior radiation treatment				
Median years	N/A	7.8	7.8	
Min/max		4.4/11.6	4.4/11.6	
Mastectomy				
Yes	8	9	17	0.360
No	1	4	5	
Adjuvant chemotherapy				
Yes	3	8	11	0.387
No	6	5	11	
Adjuvant RT				
Yes	1	1	2	0.264
No	6	12	18	

17 out of 22 patients underwent mastectomy, while wide excision was performed in 2 patients only (9.1%). Taxane-based chemotherapy was administered in 11 patients, 3 of the PAS group and 8 of the SAS group. No significant differences were noted in tumor or treatment characteristics between PAS and SAS cohorts.

**Fig. 1:**
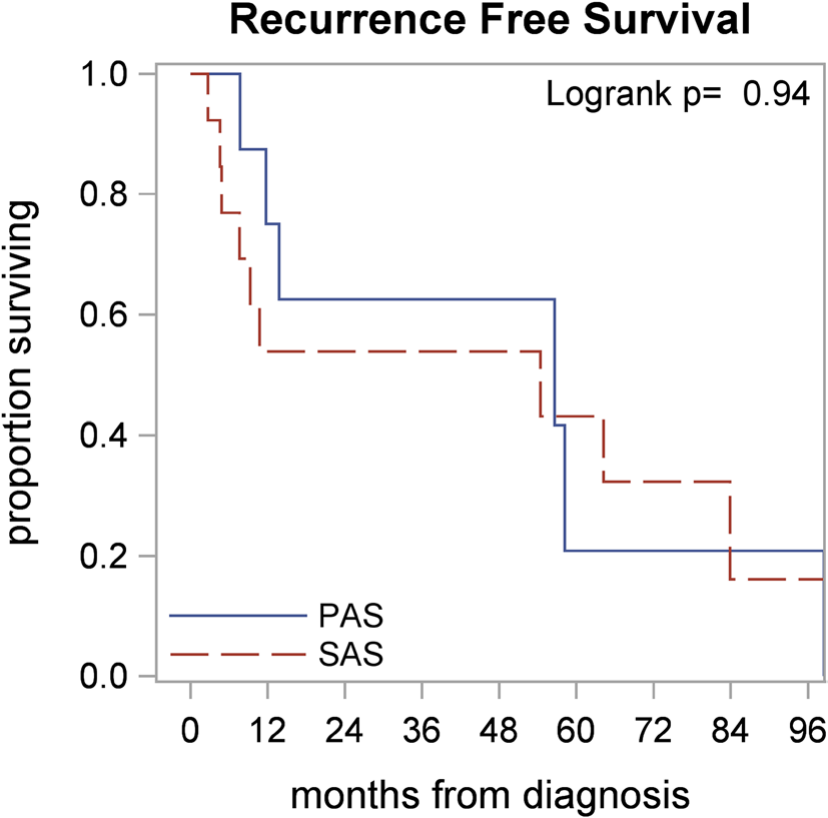
Recurrence-free outcomes by diagnosis

**Fig. 2:**
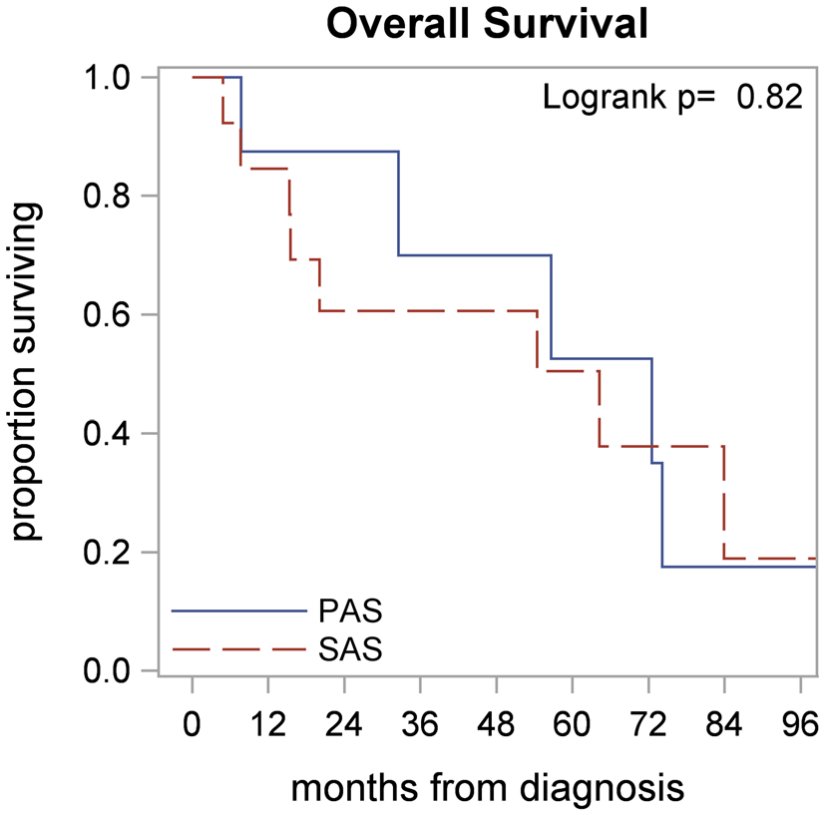
Survival outcomes by diagnosis

The patients had median follow up of 82 months from the time of their diagnosis. Recurrence-Free survival (RFS) at 3 and 5 years were 57% (95% CI 0.14–0.58) and 36% (95% CI 0.14–0.58), with estimated median RFS of 55.5 months in both groups. There were no significant differences between PAS and SAS in regards to RFS (Median RFS 56.6 vs. 54.4, *p* = 0.94), Fig. 1, The 3- and 5-year OS were 64% (95% CI 0.39–0.81) and 51% (95% CI 0.27–0.72) respectively in both groups. The PAS patients had a median OS of 72.5 months as compared to the 64.2 months for the SAS patients; however, this difference was non-significant (*p* = 0.82), Fig. 2.

**Fig. 3:**
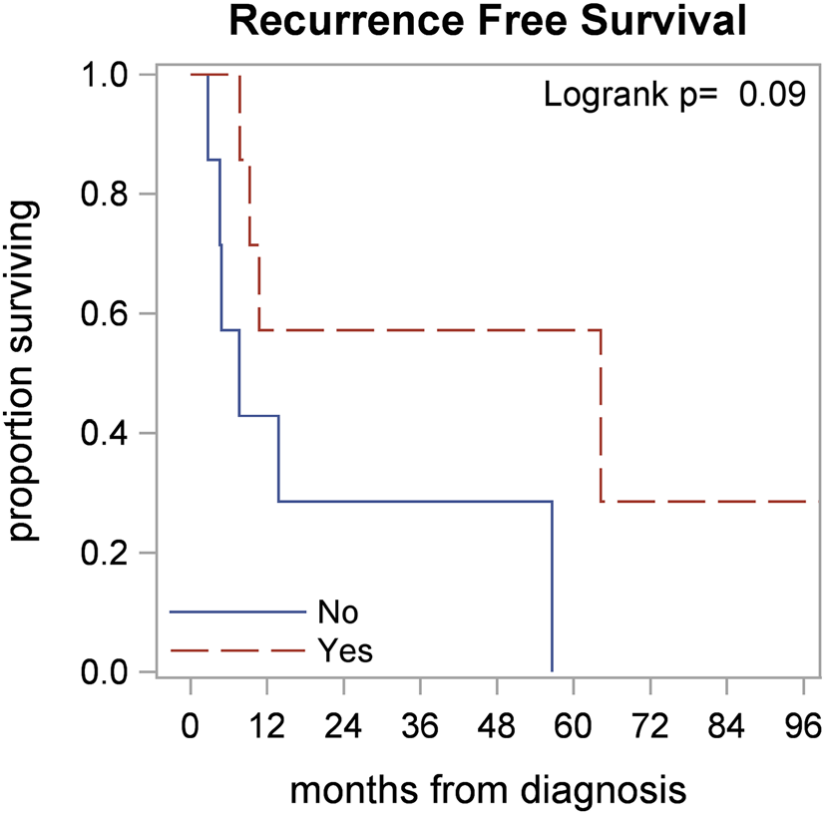
Recurrence-free survival in high grade tumors with/without chemotherapy

Mastectomy versus BCS did not seem to have a statistically significant outcome towards RFS or OS; *p *= 0.085). Furthermore, adjuvant chemotherapy and radiation therapy did not have statistically significant effects on OS or RFS. However, high grade tumors demonstrated a trend towards improved RFS when adjuvant chemotherapy was used, although this finding was not statistically significant (*p *= 0.09), Fig. 3. OS was also noted to be significantly worse in patients with lymphedema (7.6 vs. 56.6 months; *p* = 0.007), multifocal disease (15.5 vs. 64.2 months; *p* = 0.036), and African Americans (11.6 months vs. 64.2 months; *p *= 0.015). Furthermore, RFS was significantly worse in patients with lymphedema (7.6 vs. 54.4 months; *p *= 0.044) and large tumor size of > 6.9 cm (8.3 vs. 64.2 months; *p* = 0.033).

### Meta-analysis

#### Literature search results

**Fig. 4:**
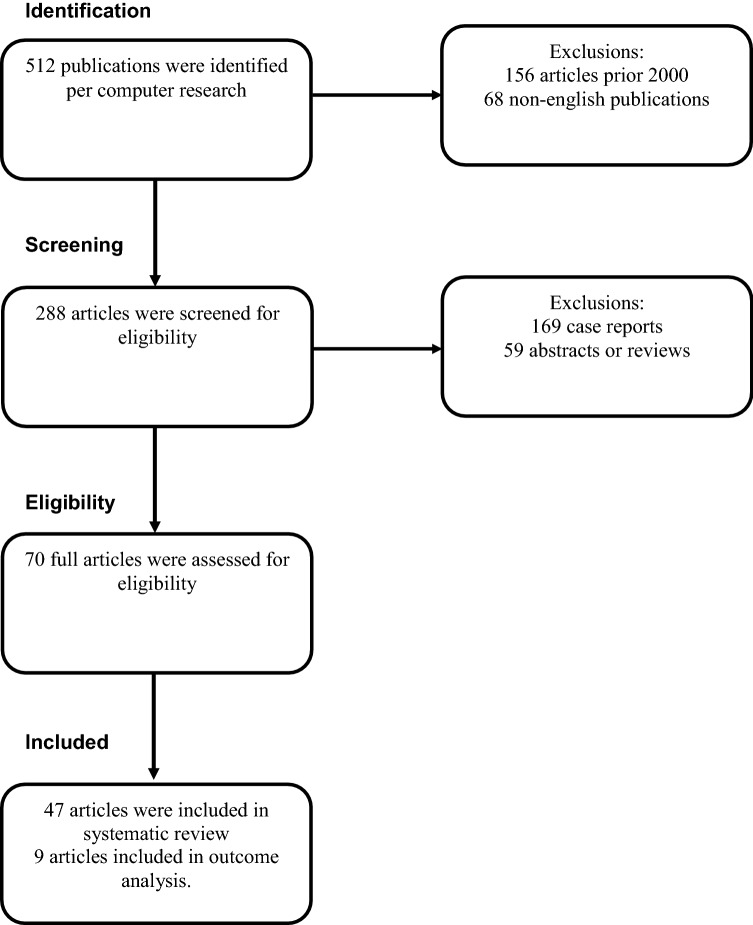
Preferred reporting items of systematic reviews and meta-analyses (PRISMA) flow diagram

512 potential articles were initially identified. 156 articles were excluded that were published prior to year 2000 and 68 articles were excluded for being in non-English language, leaving 288 articles eligible for screening. After further exclusions based on inclusion criteria, 47 eligible studies were included in the systematic review, out of which only nine articles had hazard ratio information making them eligible for survival outcome analysis. The PRISMA based study flow of the searches is presented in Fig. 4.

#### Study characteristics

Consistent with prior studies, PAS is characterized by overall younger age at presentation, averaging around 40 years compared to SAS patients who present at average age of 65 years old. PAS patients present with larger tumor size, averaging around 6 cm, whereas SAS tumors average around 4 cm in size. On average, there is a higher number of low and intermediate grade tumors in PAS in comparison to SAS who mostly presented as high grade tumors. SAS generally carry poor prognosis, and higher rates of recurrence. Median OS was observed to range between 12 and 72 months, averaging around 43 months across different studies, whereas median RFS ranging between 6 and 54 months, averaging at 18 months. PAS appear to have similar OS compares to SAS averaging around 44 months, however better RFS averaging at 26 months.

SAS histopathology appears similar in nature to other radiation associated secondary soft tissue sarcomas. Studies have demonstrated a median time averaging around 84 months to develop SAS. Most patients were managed with mastectomy and chemotherapy, and very few received radiation therapy.

#### Outcome analysis: recurrence-free survival and overall survival

**Fig. 5:**
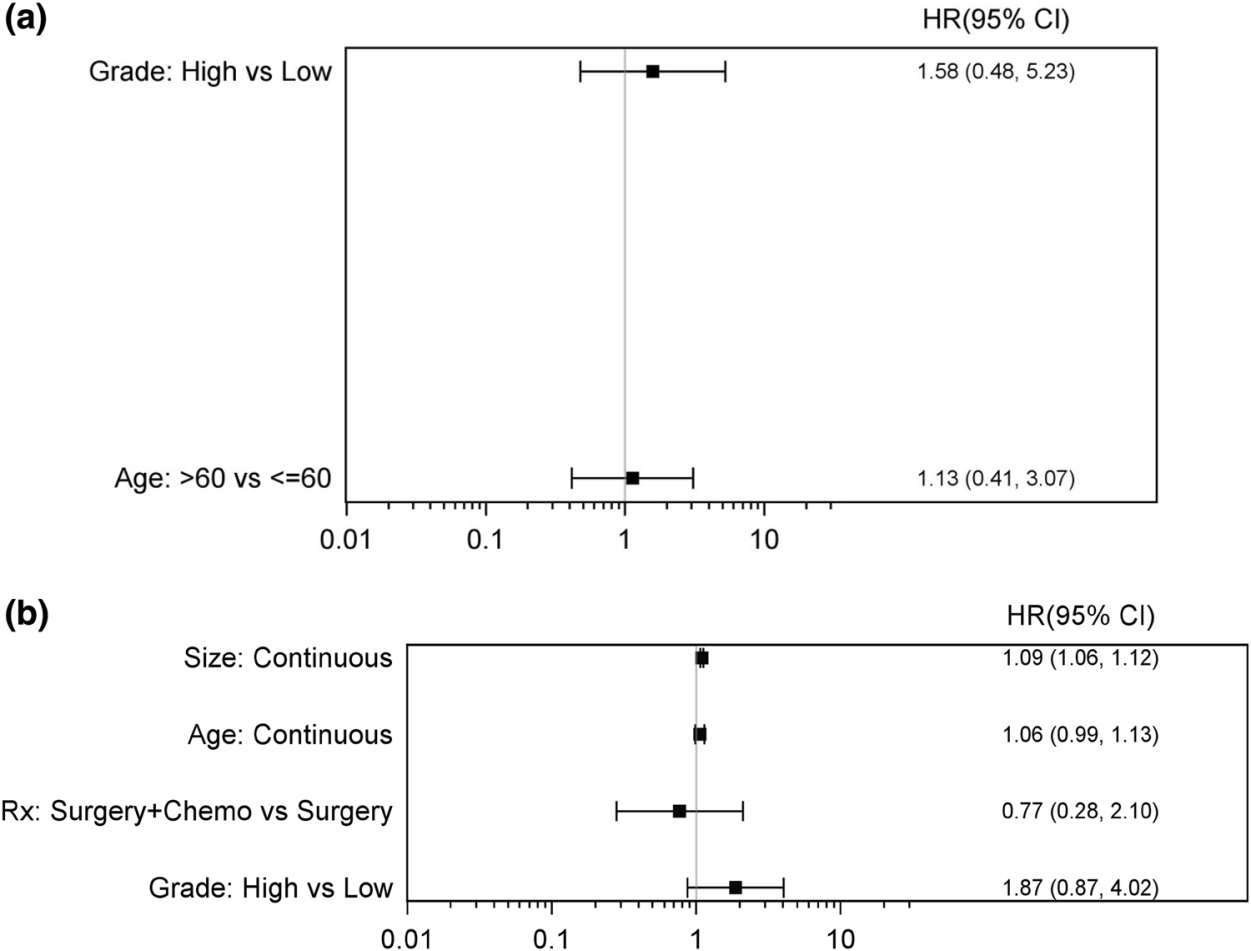
**a** Forest plot for scenario of PAS, RFS, and unadjusted. **b** Forest plot for scenario of SAS, RFS, and unadjusted

**Fig. 6:**
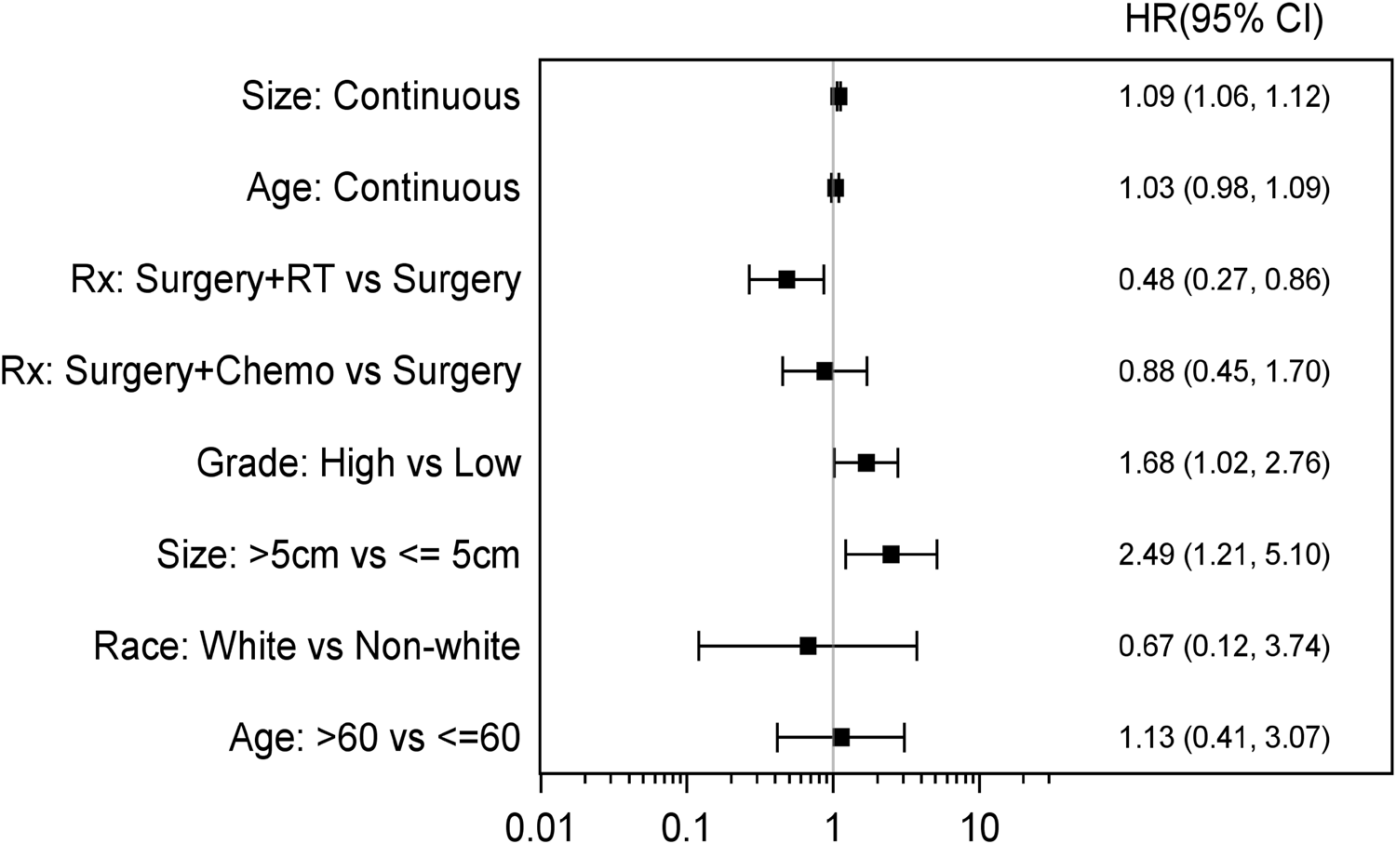
Forest plot for scenario of RFS and unadjusted

Data from 10 studies, including our retrospective cohort, were analyzed. 975 total patients were included, 380 patients with PAS and 595 with SAS. Fewer studies contained data about recurrence-free survival, 41 PAS patients and 327 SAS patients were included in the analysis. There were no statistically significant findings in the PAS group. In the SAS group, the size of tumor was significantly associated with worse RFS (HR 1.09, 95% CI 1.06–1.12) (Fig. 5a, b). Combining all PAS and SAS patients, RFS was significantly worse with high grade tumors (HR 1.68, 95% CI 1.02–2.76) and tumor size > 5 cm (HR 2.49, 95% CI 1.21–5.1). Furthermore, patients who received surgery plus RT had significantly better RFS when compared to surgery alone, suggesting that adjuvant RT may be significantly contributing to outcomes in angiosarcoma (HR 0.48, 95% CI 0.27–0.86). There were no statistically significant association between RFS and adjuvant chemotherapy. (HR 0.88, 95% CI 0.45–1.7) (Fig. 6).

**Fig. 7:**
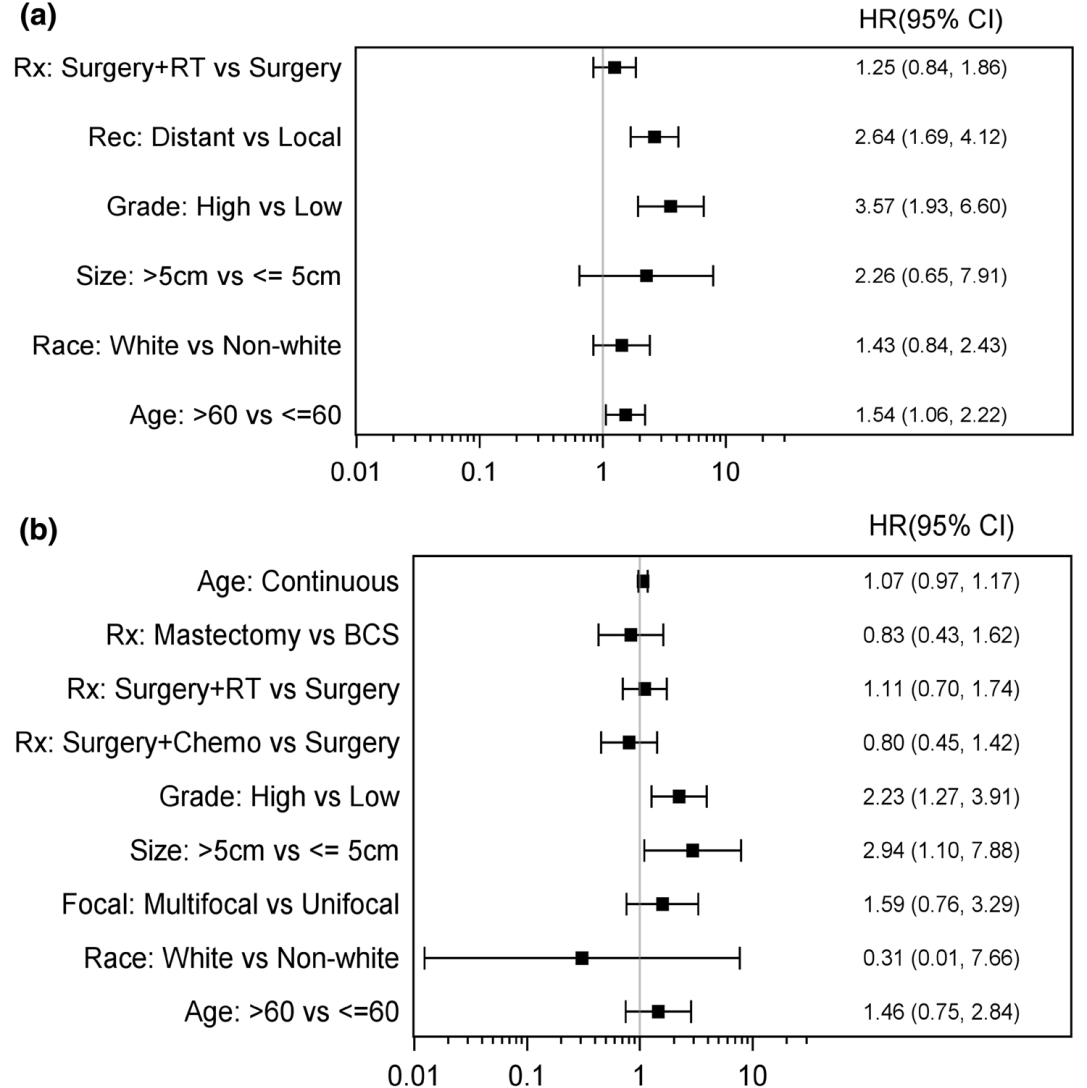
**a** Forest plot for scenario of PAS, OS, and unadjusted. **b** Forest plot for scenario of SAS, OS, and unadjusted

**Fig. 8:**
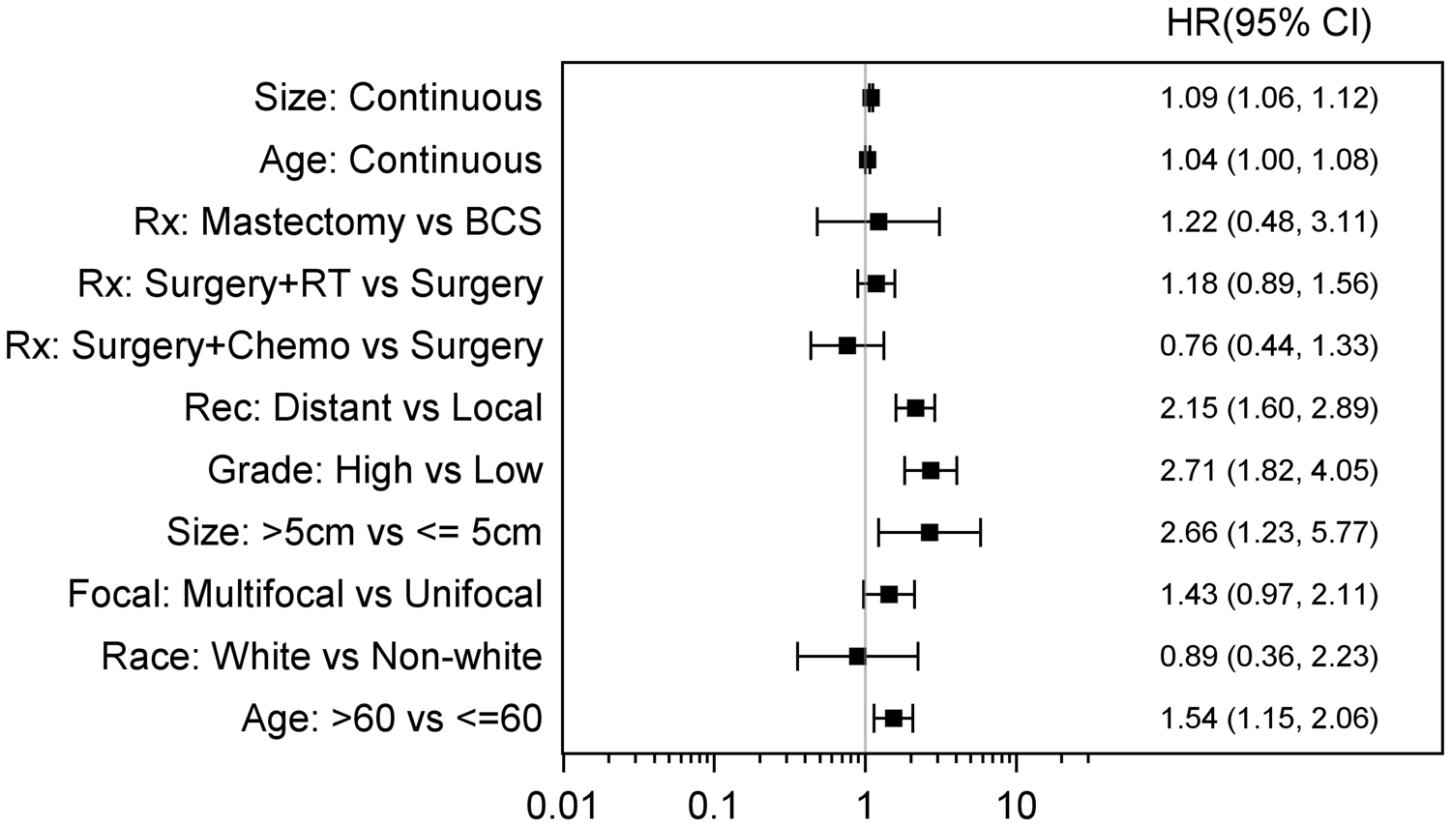
Forest plot for scenario of OS and unadjusted

In the PAS population, Age > 60, high grade and distant recurrence were all associate with significantly worse OS (Fig. 7a). In the SAS population, size > 5 cm in addition to high grade were both factors associated with significantly worse OS. Treatment approach of surgery only verses surgery plus adjuvant chemotherapy or adjuvant radiation did not seem to have a statistically significant outcome on OS in both PAS and SAS (Fig. 7b). Combining all PAS and SAS patients, OS was significantly worse with older age > 60, size > 5 cm, high grade, and presence of distant recurrence (Fig. 8). Adjuvant chemotherapy and radiation treatment had no statistically significant effects on OS. (HR 0.76; 95% CI 0.44–1.33 and 1.18; 95% CI 0.89–1.56 respectively).

## Discussion

Breast angiosarcoma is a rare and heterogeneous malignancy; therefore, most of the available literature about this disease is in the form of single institutional experiences with limited outcome data. To our knowledge, this is the largest sample size meta-analysis that is comprehensive in nature, including outcome data from both primary and secondary breast angiosarcoma, in addition to our single institutional experience.

Despite the limited literature available about breast angiosarcomas, the clinical and pathological data seem to be quite consistent across all studies. In general, SAS patients tend to be older than PAS patients, with a smaller tumor size, and generally poorer OS. Median time from radiotherapy to development of SAS varied in different reports, ranging from 51 to 180 months. Our retrospective report had a median time of 93 months, compared to the pooled literature review presented by Depla et al which reported 72 months [7].

Prognostic factors differed in impact across cohorts. Tumor size and grade seemed to be the most consistent prognostic factors for both PAS and SAS tumors in regards to both OS and RFS. Age appears to be a prognostic factor only in PAS tumors, where age > 60 was associated with worse OS. We did not find previously reported association with worse outcomes with age and interval from radiation history for SAS patients [7]. Definitive surgical resection with negative margins remains the mainstay of treatment, although the best surgical approach remains unclear. Currently, it is believed that more aggressive surgery with mastectomy concurs clinical advantage with more regional control, despite questionable survival benefit. Our retrospective data, in addition to the meta-analysis results showed no survival advantage with mastectomy. A SEER database review by Pandey et al reported that patients with PAS Grade 3 disease who were treated with surgical intervention showed no OS advantage to mastectomy vs BCS, (5-year OS 36% vs. 44%; *p* = 0.445) [11]. Yin et al’s updated SEER analysis demonstrated similar results in both PAS and SAS cohorts [10]. Furthermore, Toesca et al. also demonstrated that patients who underwent BCS did not show worse prognosis compared with those who underwent mastectomy [17]. Therefore, the question of best surgical modality for patients with breast angiosarcoma remains unsettled with R0 resection being the goal of any surgical intervention. The need for axillary lymph node dissection is unclear given nodal metastasis is not common in angiosarcoma [18].

While adjuvant chemotherapy had no survival impact in both PAS and SAS groups, adjuvant radiation therapy seemed to have a significantly positive impact on RFS when both PAS and SAS groups were analyzed together. Similar to other small studies, our retrospective study did not show any significant associations between survival and adjuvant chemotherapy or radiation therapy. However, higher grade tumors did demonstrate a trend towards improved RFS when adjuvant chemotherapy was used.

In the analysis done by Depla et al, adding adjuvant radiation therapy to definitive surgery for SAS demonstrated significant reduction in local recurrence (Local relapse free interval 57% vs. 34%, HR 0.46, *p* = 0.01) but not in OS (HR 0.87, *p* = 0.65), which is similar to the results reported in our meta-analysis. Prior studies show statistically significant benefit of adding adjuvant chemotherapy in the treatment of angiosarcomas [19], however neither our analysis nor the analysis done by Depla et al. [7] showed statistically significant benefit in breast angiosarcomas. Although, we do show that for poorer prognosis patients with high grade tumors, there was a trend toward increased benefit of RFS after administration of adjuvant chemotherapy.

Based on these results, we cannot help but speculate that mastectomy does not seem to have an additional benefit over BCS, despite seeing a more popular trend for mastectomy in these patients. Also, adjuvant radiation therapy does seem to have additional benefit in these types of tumor; however, the role of adjuvant chemotherapy remains questionable, except in high grade tumors where it seems more beneficial.

This study is limited by the rarity of breast angiosarcoma and the retrospective nature of this analysis that prevents us from drawing any definite conclusions. Some findings that failed to reach statistical significance may be due to lack of power. Furthermore, we have to take into account selection and search biases, which we tried to minimize by using two independent researchers with the final list of included studies finalized by consensus. Another limitation was the heterogeneity of the sample sizes and the reported results, with several missing data making the studies not 100% comparable, making multivariate analysis difficult.

## Conclusion

In conclusion, breast angiosarcoma is a rare aggressive tumor characterized by high grade and high rate of local recurrence. Tumor size and grade seem to be reliable predictors of survival. At this point there are no standard guidelines for treatment, with optimal R0 surgical resection and wide margins remaining to be the most agreed upon approach. Role of mastectomy verses BCS remains questionable, although mastectomy does not seem to be adding any additional benefit to BCS. Adjuvant chemotherapy does not seem to play a significant role in improving survival although taxane-based therapy can be considered in high grade and high-risk tumors. Adjuvant radiation showed statistically significant improvement in RFS and should be considered especially in cases of positive or close margins. Further studies are needed to define best treatment options for this rare tumor subtype and to define clinical differences in PAS versus SAS.

## Data Availability

The datasets used and/or analyzed during the current study are available from the corresponding author on reasonable request.
